# Investigation of the value of a photographic tool to measure self-perception of enamel opacities

**DOI:** 10.1186/1472-6831-12-41

**Published:** 2012-10-09

**Authors:** Gill M Davies, Iain A Pretty, Janet S Neville, Michaela Goodwin

**Affiliations:** 1 The Dental Observatory, c/o Central Lancashire PCT, Jubilee House, Centurion Way, Leyland, PR26 6TR, UK; 2 Dental Health Unit, School of Dentistry, University of Manchester, Lloyd Street North, Manchester Science Park, England, M15 6SH, UK

**Keywords:** Enamel opacities, Self-perception, Epidemiological tool

## Abstract

**Background:**

The standard measurement of oral conditions that are mainly of cosmetic concern can be carried out by a trained clinical professional, or they can be assessed and reported by the individuals who may have the condition or be aware of others who have it. Enamel opacities of anterior teeth are examples of such a condition. At a public health level the interest is only about opacities that are of aesthetic concern, so the need for an index that records opacities that the public perceive to be a problem is clear. Measurement methods carried out by highly trained professionals, using unnatural conditions are not indicated at this level. This study reports on the testing of a novel epidemiological tool that aims to report on the prevalence and impact of self-perceived enamel opacities in a population of young adolescents.

**Methods:**

A dental health survey was carried out using a random sample of 12-year-old school pupils during 2008/09 by Primary Care Organisations (PCOs) in England. This included the use of a novel self-perception tool which aimed to measure individual’s self-perception of the presence and impact of enamel opacities to produce population measures. This tool comprised questions asking about the presence of white marks on their teeth and whether these marks bothered the volunteers and a sheet of grouped photographs of anterior teeth showing opacities ranging from TF 0, TF 1–2 to TF 2–3. Volunteers were asked which of the groups of photographs looked more like their own teeth. Examining teams from a convenience sample of 3 PCOs from this survey agreed to undertake additional measurements to assess the value of the self-perception tool. Volunteer pupils were asked the questions on a second occasion, some time after the first and clinical examiners recorded their assessments of the most closely matching set of photographs of the volunteers on two occasions.

**Results:**

The tool was feasible to use, with 74% of pupils making a response to the first question about the presence of white marks on front teeth, 94% to the second (do these marks bother you?) and 79% to the third about which set of images most closely matched the volunteer’s own, with regard to white marks. Responses to these sequential questions showed coherence with pupils who perceived themselves as having white marks on their teeth being more likely to select images that showed teeth with opacities to match with their appearance. Pupils who reported themselves concerned about their white marks were the most likely to select images with the most severe opacities. Repeatability was good among pupils (Kappa = 0.65) and very good among examiners (Kappa = 0.87). Agreement levels between pupil’s and examiner’s choice of images was poor as examiners were less likely than pupils to select images that showed more severe levels of mottling.

**Conclusions:**

With regard to feasibility, coherence and repeatability the standardised epidemiological tool under scrutiny, with operator training, appears to be a suitable method for measuring the prevalence and impact of self-perceived enamel opacities in a population of young adolescents.

## Background

Dental fluorosis has been measured using a wide range of methods, the majority of which are undertaken and reported by dental professionals [1-5]. Such methods often involve drying and illuminating teeth, photographing them and examining them at high levels of magnification against a range of clinical indices [6-8]. These techniques tend to reveal higher levels of prevalence than seen in their natural state and they include very mild levels of opacities that are potentially of little aesthetic concern at a public health or individual level. They may be relevant in clinical trials and epidemiological studies but, as they ignore the subject’s voice, they are of little value in ascertaining the impact of dental fluorosis as a public health issue [9-13].

The use of clinical indices such as the DDE, TF and Dean’s in epidemiological studies has been widely reported [8,14-16]. While each index has strengths and weaknesses, a common issue is the complexities in training and calibration for measurement of opacities [8,16] leading to low reported agreement values between examiners, resulting in many studies being undertaken by a single examiner. The detection of mild white lines and patches on anatomical structure that are themselves white is prone to confounding issues of lighting, hydration status of the teeth, visual acuity of the examiner, angulation of viewing and the presence of plaque [17]. Such indices are based on normative standards and do not include an assessment of the subject’s own view of their teeth or their appearance [18]. Therefore using measures gained solely by clinical professionals could be seen as unreliable given the difficulty to train and calibrate on a given index.

The presence of enamel opacities, including fluorosis, is only a public health problem if the public perceive it to be so. Several studies have shown that the milder levels of fluorosis are not regarded as being of aesthetic concern [18], indeed some studies have shown a preference for it compared with teeth with no opacities [19]. Therefore it is important to go to the source - the individual, to ascertain the impact of enamel opacities in an epidemiological setting.

The Water Act (2003) [20] requires that the health of persons living in the area being fluoridated should be monitored and this requirement might be expected to include the levels of fluorosis. A robust method is required whereby the levels of enamel mottling can be recorded with ease in a population and this may be done as part of the NHS Dental Epidemiology Programme in England. A method that records the perceptions of enamel opacities, and its impact upon individuals in the population would seem to be more appropriate than a measure which relates only to a normative assessment.

A simple tool was therefore developed to capture what people thought about their own teeth in an epidemiological setting. This study aimed to determine the effectiveness of using this tool to assess self reported prevalence and impact on an individual of enamel opacities in an epidemiological survey.

## Methods

As part of the NHS Dental Epidemiology Programme for England all Primary Care Organisations (PCOs) were required to undertake dental health surveys of a random sample of 12-year-old school pupils during 2008 / 09 according to a national protocol. These surveys took place under the epidemiological programmes national ethical approval (Department of Health, England) and required parental opt out consent and positive consent from the participating children. The surveys took place in mainstream, state funded schools and involved sample sizes of a minimum of 250 pupils for each Local Authority and PCO. The purpose of these surveys was to measure the prevalence and severity of caries, the need and demand for orthodontic care, impact of oral disease and reported self-care. Additionally, for the first time, two questions and a photographic tool were used to collect information about self-perception of the presence and impact of enamel opacities. In this section of the survey the first question asked was “Do you have any white marks on your front teeth that won’t brush off?”. A second question was asked of those who replied “Yes” to the first; “Does the appearance of these marks bother you?”.

**Figure 1:**
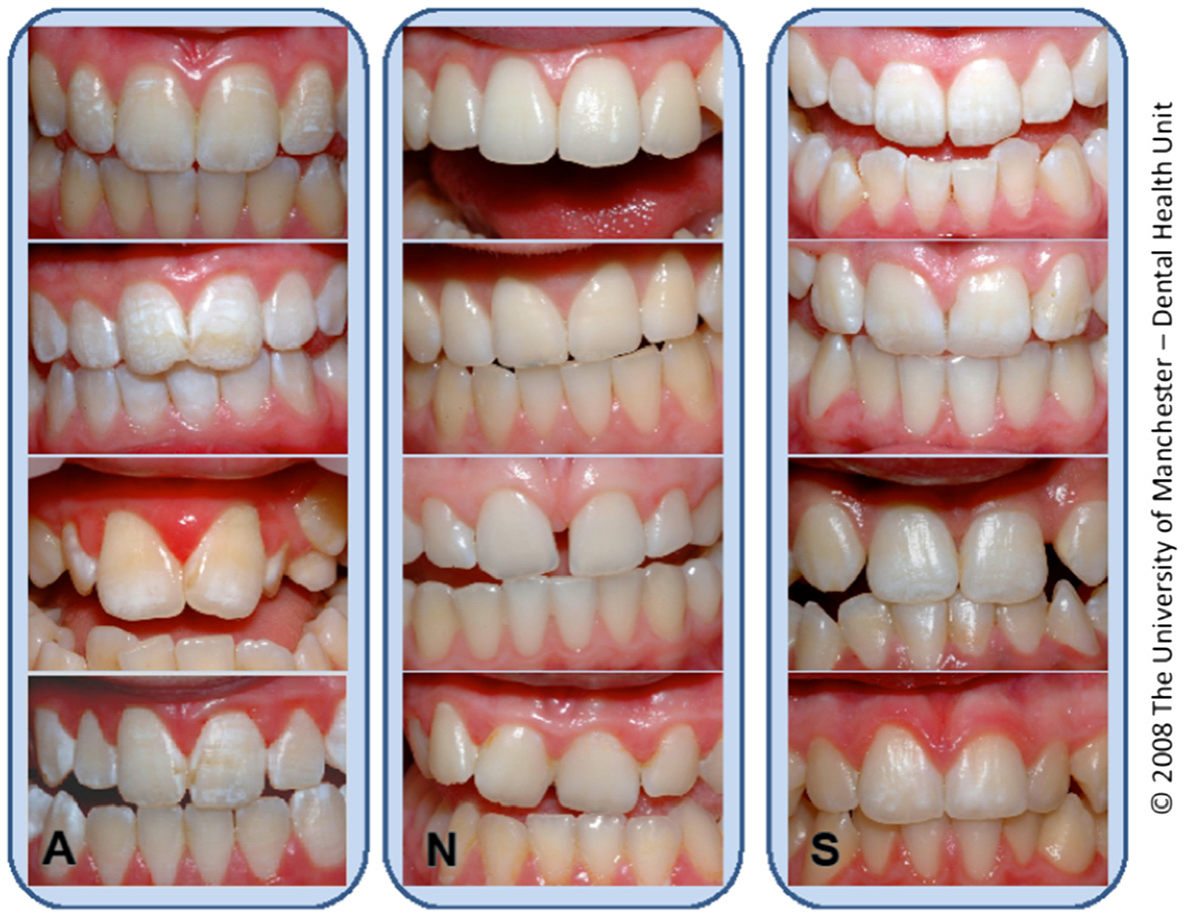
**The self-perception tool as supplied to the examiners and volunteers.** Reproduced with the permission of the copyright holders.

In order to measure the self-perception of any enamel opacities each volunteer was then shown a sheet of grouped photographs illustrating the typical appearance of teeth that had no opacities (TF = 0), mild opacities (TF = 1-2) and moderate opacities (TF = 2-3) (Figure 1). These photographs showed just the anterior teeth, with lips retracted, teeth dried, with good illumination and some minor magnification. As the photographic appearance of enamel opacities can vary with minor differences in duplication methods a standard print setting was established and the quality of each image checked against a standard image. For this reason photocopied or self printed versions of the self-perception tools were not permitted. The sets of four photographs of each fluorosis type were grouped vertically together and the groups were ordered randomly across the page. Labels for each set were chosen and used in such a way to avoid any ranking or hierarchy. All the volunteers were asked “Thinking about white marks on teeth, do you think your front teeth look more like those in this group, or the ones in this group, or this group?” while the interviewer indicated each strip of grouped photographs, in random order.

No mirrors were used as it was considered that those volunteers who were concerned about, or aware of, their enamel opacities would know how they looked. Those who had not noticed them previously were, *de facto*, not concerned about their dental appearance.

For the purpose of assessing the repeatability and use of the self-perception tool in this setting a convenience sample of three Primary Care Trusts (PCOs) agreed to carry out additional measures about enamel opacities, all in non-fluoridated areas. In two PCOs the examining dentist looked at the upper anterior teeth of each volunteer and indicated which set of photographs in the self perception tool they considered most closely matched the appearance of the volunteer with regard to enamel opacities. All three examining teams included the self-perception questions and photographic tool again when they undertook repeat examinations to test for repeatability of the enamel opacity data. To achieve this, randomly selected volunteers were asked to return at the end of an examining session so that a brief version of the overall survey could be repeated. For this second examination the sets of images on the self-perception tool were re-ordered and re-labelled so that the respondent could not simply remember which set they chose at the first time of asking. These additional data allowed for assessments of consistency and repeatability.

Data were entered into SPSS, descriptive summaries were provided for answers relating to self-perception of white marks on the teeth, for photographs selected. Analyses assessing inter-rater agreement between volunteers and examiners and intra rater agreement were conducted using the kappa statistic.

## Results

The teams in three PCOs took part in additional activities to measure the effectiveness of the self-perception tool with regard to feasibility, coherence and repeatability (See Table 1). A sample of 2,803 pupil volunteers aged 12 years, were involved from the main NHS Dental Epidemiology Programme 2008/09 survey. Not all volunteer pupils were involved in all stages of the exercises as not all examiners coded the pupils against the tool (Table 2).

**Table 1:** Activities by each PCO

**PCO**	**1**^**st **^**examination – volunteer’s perception**	**Repeat examination – volunteer’s perception**	**1**^**st **^**examination – examiner’s perception**	**2**^**nd **^**examination – examiner’s perception**
Manchester	Y (417)	Y (26)	N (0)	N (0)
Central Lancashire/ Warrington	Y (2386)	Y (215)	Y (2303)	Y (262)

**Table 2:** Selection of images showing a range of TF scores by response to questions about white marks (N = 2800*)

	**Set of photographs selected by volunteers**			**Don’t know**	**Total**
	**TF = 0**	**TF = 1 - 2**	**TF = 2 - 3**		
All volunteers	1,581 (56%)	470 (17%)	256 (9%)	493 (18%)	2800
Those who self reported having white marks	131 (33%)	111 (28%)	85 (21%)	70 (18%)	397
Those who reported no white marks	1065 (63%)	247 (15%)	104 (6%)	271 (16%)	1687
Those whose self reported white marks bothered them	21 (22%)	32 (34%)	25 (27%)	16 (17%)	94
Those who reported not bothered by white marks	97 (35%)	74 (27%)	56 (20%)	49 (18%)	276

*3 participants did not provide an answer.

### Responses to questions

**Figure 2:**
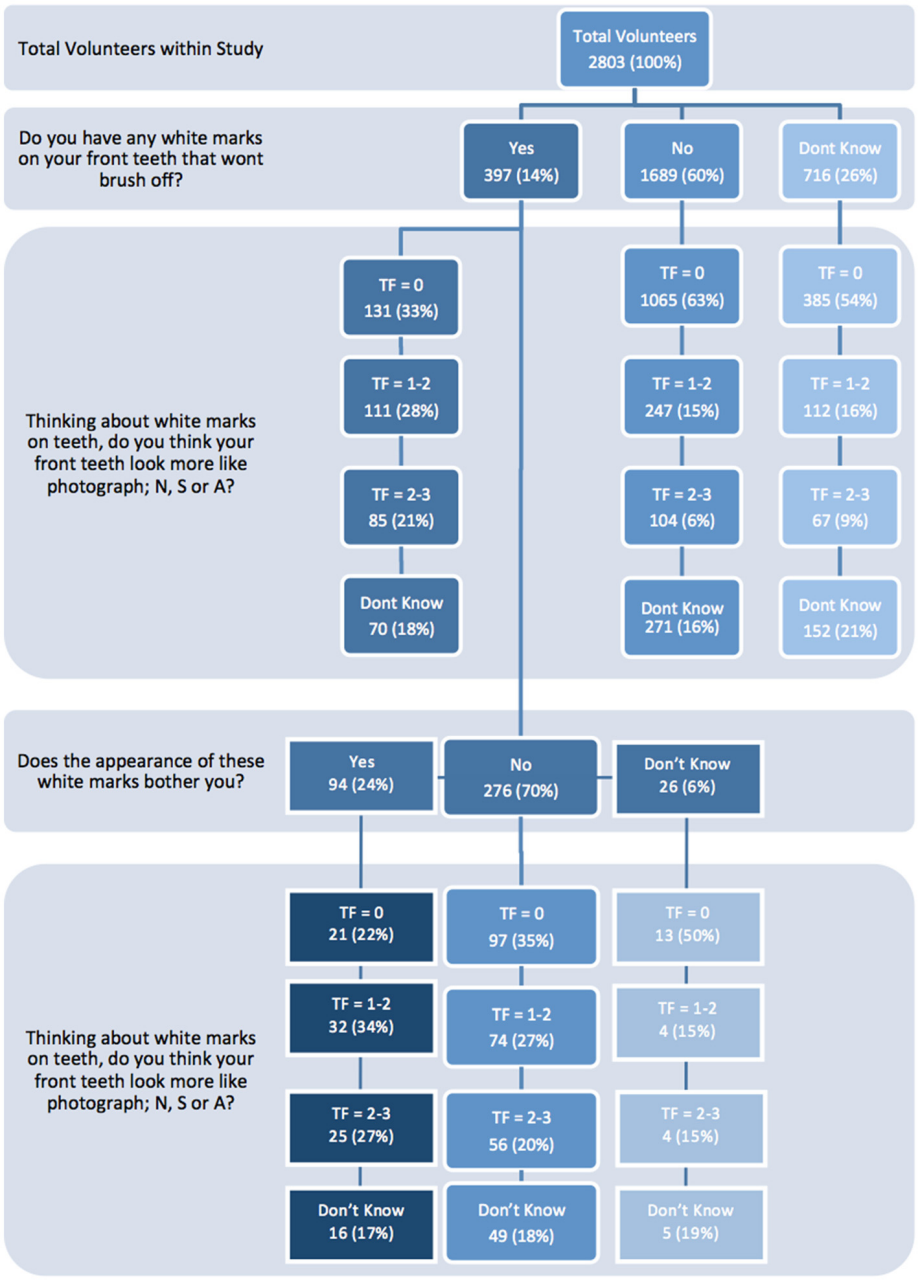
**Flow of patient’s responses.** *One participant did not answer throughout questions.

When asked in the main survey “Do you have any white marks on your front teeth that won’t brush off?” 397 out of 2,803 (14%) volunteers responded ‘yes’ (Figure 2). Of these, 94 (24% of those asked and 3.4% of the total) replied ‘yes’ to the follow-on question “Does the appearance of these marks bother you?”.

### Selection of images

When asked to indicate which set of photographs they thought most closely matched the appearance of their front teeth with regard to white marks 1,581 (56%) indicated the set with no enamel opacities, 470 (17%) indicated mild enamel opacities and 256 (9%) the set with moderate opacities (Table 2). A response of ‘don’t know’ was given by 493 (18%) volunteers.

Among those who had self-reported having white marks there was an increased proportion who selected the images depicting more obvious opacities; 28% selecting images showing TF 1–2 and 21% selecting TF 2–3. This trend was seen more acutely among those who stated that their self reported white marks bothered them; 34% of these selected images of TF 1–2 as being most like the appearance of their own teeth and 27% selecting the TF 2–3 set. Similar proportions of volunteers in these sub groups replied ‘don’t know’ when asked to select a matching set of images. This is shown in Figure 2 which demonstrates the flow of the decisions and the selections made.

### Selection of images by dental examiners

The examining dentists in two of the PCOs recorded their selection of photographs that most closely matched the appearance of 2,302 volunteers. Table 3 compares the selections made by the examiners and those made by the volunteers about themselves. The examiners considered that the images that showed no enamel opacities were the closest match for 1,473 (77%) of the volunteers that they assessed. They considered that the photographs showing mild opacities matched with 356 (19%) of volunteers and the images of more marked opacities matched 84 (4%). They were more likely to select the TF 1–2 images for those pupils who had reported that they had white marks on their teeth and more likely to select the TF 2–3 for those reporting being bothered by their white marks, when comparing this to examiner selections for all volunteers.

**Table 3:** Selection of images showing varying severity of enamel opacities by both examiners and volunteers (N = 1913*)

	**Images showing TF = 0**		**Images showing TF 1-2**		**Images showing TF 2-3**		**Response ‘don’t know’**	
	**Examiner**	**Volunteer**	**Examiner**	**Volunteer**	**Examiner**	**Volunteer**	**Examiner**	**Volunteer**
All volunteers (n = 1913)	1473	1336	356	362	84	215	-	389
	77%	70%	19%	19%	4%	11%		17%
Those who self reported having white marks (n = 263)	114	108	106	85	43	70	-	51
	43%	41%	40%	32%	16%	27%		19%
Those whose self reported white marks bothered them (n = 63)	29	17	19	25	15	21	-	13
	46%	27%	30%	40%	24%	33%		17%

*sample includes cases where both the examiner and volunteer gave a response, excluding those who responded ‘don’t know’ and ‘not answered’.

The volunteers who made a selection were more likely than examiners to select the images that showed the most marked opacities (11% vs. 4%) and less likely than the examiners to indicate the images that showed no opacities (70% vs. 77%). These trends of selecting higher severity images continued amongst those who reported that they had white marks on their own teeth and were concerned by the appearance of them (33% vs. 24%).

When the selections of images made by the volunteers and the selections about the same volunteers made by the examiners are compared there is a poor level of agreement (Kappa = 0.10) (Table 4). In 62% of cases there was agreement between the volunteer’s and the examiner’s selection of images. There was a disparity of 1 degree either way in 30% of cases, leaving 8% of cases where there was clear disagreement between the two assessments.

**Table 4:** Examiner assessment of enamel opacities, by self-perception of the same (N = 1913)

**Set of photographs selected by examiner**	**Set of photographs selected by volunteers**			**Total**
	**TF = 0**	**TF = 1 - 2**	**TF = 2 - 3**	
TF = 0	1,083	259	131	1,473
TF = 1 - 2	227	75	54	356
TF = 2 - 3	26	28	30	84
Total	1,336	362	215	1,913

kappa = 0.10, Weighted kappa = 0.14.

### Repeat assessments

Repeat examinations of the pupil self-perceptions indicate that repeatability of the tool is ‘good’ with a Kappa value of 0.65 (Table 5). In 83% of cases the same set of images were selected on both occasions, in 14% there was a disparity of 1 level and in 3% a disparity of two levels. Repeat examinations of the examiner classification indicate that reproducibility of the tool is ‘very good’ with a Kappa value of 0.87 (Table 6). In 95% of cases the same set of images were selected on both occasions and in 5% there was a disparity of 1 level.

**Table 5:** Repeat assessment of self-perception of enamel opacities by volunteers

**Set of photographs selected by volunteer at first examination**	**Set of photographs selected by volunteers at repeat examination**			**Total**
	**Image set K TF = 0**	**Image set B TF = 1 - 2**	**Image set Y TF = 2 - 3**	
Image set N	143	14	1	158
TF = 0				
Image set S	12	30	2	44
TF = 1 - 2				
Image set A	4	5	18	27
TF = 2 - 3				
Total	159	49	21	229

(N = 229).

Kappa = 0.65 = good agreement.

**Table 6:** Repeat assessment of self-perception of enamel opacities by examiners

**Set of photographs selected by examiners at first examination**	**Set of photographs selected by examiners at repeat examination**			**Total**
	**Image set K TF = 0**	**Image set B TF = 1 - 2**	**Image set Y TF = 2 - 3**	
Image set N	183	3	0	186
TF = 0				
Image set S	9	46	1	56
TF = 1 - 2				
Image set A	0	1	17	18
TF = 2 - 3				
Total	192	50	18	260

(N = 260).

Kappa = 0.87 = Very Good agreement.

## Discussion

The purpose of this study was to assess a simple, visual tool to determine if it could measure a child's own perception of the appearance of their teeth – and in particular their own of enamel opacities. The results clearly demonstrate (see Table 2) that there is an association between the self-perception of the presence of opacities and the choice of images from the tool, in the direction one would expect. Children who consider themselves to have white marks on their teeth are more likely to select those photographs with more severe representations of mottling. The same coherence was found among the examiners’ measurement using the same tool but at a lower level of severity than for the volunteers’.

Repeatability appeared to be good with regard to selection of images for pupils and very good for examiners. With only three sets of images from which to select and with very little difference between them it might have been expected that the volunteers would simply pick a set of images at random but the Kappa score of 0.65 for repeat examinations shows that this was not the case. Changing the order and the labelling of the grouped images for the second time of questioning ensured there could be no accusation of simply picking the images in the same position as the first occasion.

When considering individual responses there was poor agreement [21] between the examiner’s selection of images and those of the individual volunteers. This illustrates the concerns raised about other indices of enamel opacities which are measured by trained, clinical professionals and which may over- or under-estimate the prevalence, severity and *impact* of enamel opacities [22]. Years of training and an underlying understanding of the biological basis for enamel opacities among examiners is in contrast to the subjective (but authentic) view of an individual based on their feelings about appearance, aesthetics and acceptability [23]. An additional point is that individual variances between examiners and pupil volunteers are unlikely to be of importance within epidemiological surveys where data are reported at group level and the purpose of such studies is to gain a view on population impact. Using responses from individual volunteers in this way is further supported when one considers the good repeatability scores achieved by the subjects when using this tool [24].

In the national survey similar results were found to those reported here for a subset of PCOs [25]. Some variations between PCOs were found which suggest some results should be viewed with caution. For example, in one or two PCOs an unusually high proportion of pupils were reported to not know which images to select as matching with their own appearance and in other PCOs a high proportion chose not to answer one or more of the self-perception questions. This might suggest that there has been unusual administration of the tool that has lead to volunteers not engaging with the activity. This may be an issue that can be addressed through better training to ensure improved compliance with the correct method of eliciting responses.

While this study attempted to reduce the various biases that can occur in this type of survey, such as selection bias by using a random sample, order bias by scrambling the arrangement of photos to be chosen by volunteers, there are some areas that could not be lessened. For example, an issue within this type of survey is that simply asking a question about white marks may incline a participant to consider this when they never had before. Additionally, in regards to the limitations of this study, as it is purely a descriptive observational survey it cannot be used to determine the construct validity of the tool at this stage, especially given the lack of an agreed reference standard. Qualitative work would be required to gain further understanding of the different selections made and to establish if this tool really reflects how the participant feels regarding the aesthetic appearance of their teeth in relation to enamel opacities.

Tools used in epidemiological studies should be simple, efficient, and economical. They should not require complicated or expensive equipment and should be impervious, if possible, to the range of settings within which such studies are undertaken. The tool described within the current work meets these objectives and can be utilised in multiple sites simultaneously and by a number of epidemiological teams within the same site. The tool was found to be easy to implement and understood by the volunteers and requires limited training and no calibration of examiners.

## Conclusions

This tool, comprising three questions and a standard set of photographs, appears to fulfill the requirements of an effective epidemiological tool with regard to feasibility, coherence and repeatability and is suitable for the measurement of self reported prevalence of enamel opacities and the impact they have in a population of young adolescents.

## Competing interests

MG and IAP work within the Dental Health Unit. This Unit receives an unrestricted grant from Colgate Palmolive.

## Author’s contributions

GD and IAP devised the protocol and the standardised images, both contributed to writing the final manuscript. GD integrated the study into the national epidemiological programme and supported its implementation. JN entered the data and provided initial statistical support. MG undertook the analysis of the data and assisted in the preparation of the manuscript.

## Pre-publication history

The pre-publication history for this paper can be accessed here:

http://www.biomedcentral.com/1472-6831/12/41/prepub
